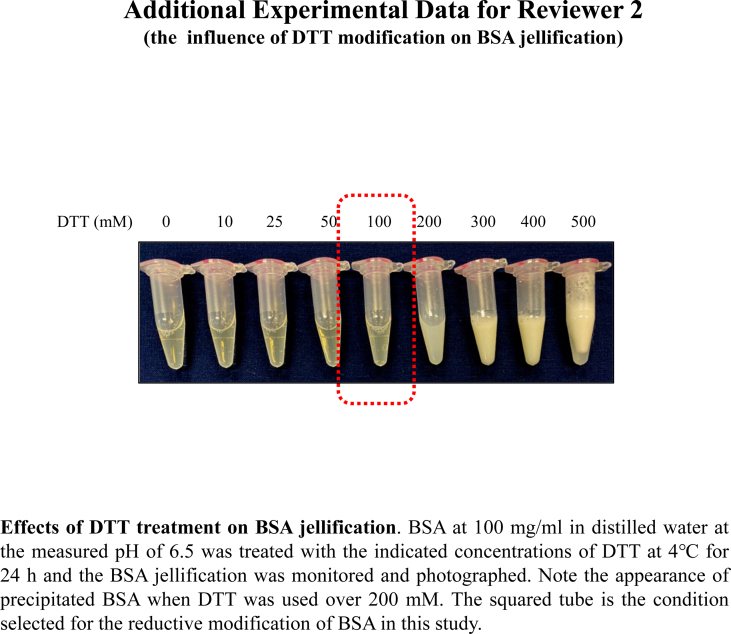# Reductively modified albumin attenuates DSS-Induced mouse colitis through rebalancing systemic redox state

**DOI:** 10.1016/j.redox.2021.101881

**Published:** 2021-02-05

**Authors:** Xiawen Yang, Zhimin Mao, Yanru Huang, Haizhao Yan, Qiaojing Yan, Jingru Hong, Jianglin Fan, Jian Yao

**Affiliations:** a Divison of Molecular Signaling, Department of the Advanced Biomedical Research, Interdisciplinary Graduate School of Medicine, University of Yamanashi, Chuo, Yamanashi, Japan; b Department of Molecular Pathology, Interdisciplinary Graduate School of Medicine, University of Yamanashi, Chuo, Yamanashi, Japan

**Keywords:** Colitis, Albumin, Reductively modified albumin, Sulfenic acid formation, Hydrogen peroxide, Oxidative stress

## Abstract

Albumin (Alb) is the most abundant plasma protein with multiple biological functions, including antioxidative property through its thiol activity. Given that inflammatory bowel disease is associated with a decreased level of Alb and an increased level of Alb oxidation, we asked whether Alb could have a therapeutic effect on colitis. Here we tested this possibility. Bovine serum albumin (BSA) was reductively modified with dithiothreitol (DTT) and administrated via gavage or intraperitoneal injection. Dextran sulfate sodium (DSS)-induced mice colitis was associated with massive oxidative stress, as indicated by the elevated sulfenic acid formation in blood, colon tissues, and feces. Treatment of mice with the reductively modified albumin (r-Alb) attenuated the oxidative stress and reduced local inflammation and tissue injury. These effects of r-Alb were only partially achieved by unmodified Alb and wholly lost after blocking the –SH groups with maleimide. In cultured colon epithelial cells, r-Alb prevented DSS- and H_2_O_2_-induced ROS elevation and barrier dysfunction, preceded by inhibition of sulfenic acid formation and P38 activation. Further analysis revealed that Alb was susceptible to H_2_O_2_-induced oxidation, and it detoxified H_2_O_2_ in a –SH group-dependent way. Moreover, Alb reacted with GSH/GSSG via thiol-disulfide exchange and reciprocally regulated the availability of –SH groups. Collectively, our study shows that r-Alb effectively attenuates DSS colitis via –SH group-mediated antioxidative action. Given that the oxidative stress underlies many life-threatening diseases, r-Alb, functioning as a potent antioxidant, could have a wide range of applications.

## Highlights

• Albumin, the major thiol antioxidant in body, is decreased and oxidized in colitis.
• Reductive modification of albumin increased its thiol-antioxidative activity.
• Modified albumin ameliorated DSS colitis and improved systemic redox state.
• Modified albumin detoxified H_2_O_2_ and prevented H_2_O_2_-induced cell injury.
• Modified albumin interacted with GSH/GSSG/H_2_O_2_ via sulfhydryl group.

## Graphical abstract

**Figure:**
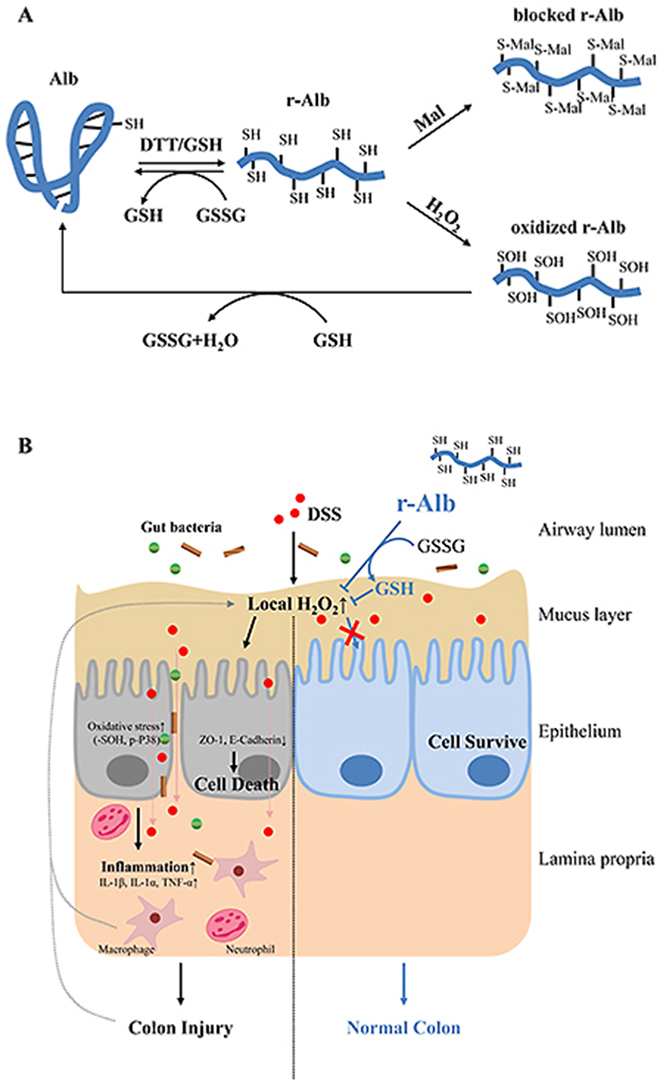

## 1 Introduction

Inflammatory bowel disease (IBD) afflicts millions of people worldwide. It refers to conditions with chronic inflammation of the gastrointestinal tract. Two common forms of IBD in the clinic are ulcerative colitis (UC) and Crohn's disease. The clinical characteristics of IBD include abdominal pain, blood in the stool, diarrhea, and weight loss, which greatly influence the quality of life of the patients [1]. Currently, the therapy involves immune suppression with steroids and immunomodulators, as well as surgery. These therapies, however, cannot lead to a long-lasting remission and cure of the diseases. Therefore, it is highly desirable to under the molecular mechanisms underlying IBD and find more effective therapeutic strategies.

Dextran sulfate sodium (DSS)-induced mice colitis is a well-used animal model to investigate the pathogenesis of IBD [2,3]. Mice with DSS colitis have similar signs and symptoms to human UC, such as weight loss, diarrhea, mucosal ulceration, and shortened colon length. The mechanisms involved in DSS colitis include the increased production of reactive oxygen species (ROS), dysfunction of the mucosal defense, activation of local inflammatory responses, and induction of intestinal cell injury [[4], [5], [6], [7], [8]]. Among them, a large amount of ROS released by the inflammatory cells plays a pivotal role [9,10]. It contributes to many pathological changes in intestinal tissues and is closely correlated with the disease activity. Growing evidence indicates that antioxidative therapies are useful in the treatment of IBD [[11], [12], [13]].

Oxidative stress is resulted from the imbalance between the production of ROS and its removal by the detoxifying mechanisms of biological systems. It has been recognized as a common mechanism of many life-threatening diseases, including colitis [[13], [14], [15], [16], [17]]. ROS accumulation causes oxidative cell responses through oxidative modification of macromolecules and activation of redox signaling pathways. As an integral part of the body defense system against oxidative stress, thiol antioxidants participate in coordinating the antioxidative actions and maintaining the redox balance. Free sulfhydryl (-SH) groups neutralize the oxidants to a less toxic by-product through reversible thiol-disulfide exchange reaction. It controls cell function and cell fate [18].

Albumin (Alb) is the most abundant plasma protein in human blood, accounting for 50%–60% of total plasma proteins in healthy adults. Alb has several critical physiological functions. It regulates osmotic pressure, influences fluid distribution, and transports metals, fatty acids, cholesterol, bile pigments, and drugs [19]. Besides, Alb is the major antioxidant within body fluid [20,21]. Structurally, Alb is a single polypeptide of 585 amino acids. It contains 35 cysteine residues, which forms 17 intramolecular disulfide bonds and leaves Cys34 as a –SH group. Because Alb is the most abundant protein in the blood, its Cys34 is also the largest source of free thiol antioxidant within body fluid [[22], [23], [24], [25]]. Apart from Cys34-mediated direct antioxidative actions, Alb also has indirect antioxidant activity through binding molecules that are critically involved in redox reactions, such as metals, oxysterols, bilirubin, and homocysteine. Alb is also highly susceptible to oxidative modifications, such as thiol oxidation and carbonylation [22,23,[26], [27], [28], [29]]. In humans, serum Alb exists in both reduced (mercaptalbumin) and oxidized forms (non-mercaptalbumin) [30]. In reduced Alb, the reactive –SH group Cys34 is free, whereas, in oxidized Alb, it forms mixed disulfide bonds with other molecules or exists as oxidized products. The redox status of its cysteine residues in Alb is influenced by the oxidants in the environment and reflects the systemic oxidative stress condition.

In both experimental model and IBD patients, the serum level of Alb is reported to be decreased, whereas the oxidized Alb is increased [22,[31], [32], [33]]. Furthermore, the altered Alb is closely correlated with inflammatory activity and has been proposed as prognostic marker of UC diagnosis [31]. In these backgrounds, we, therefore, hypothesized that the supplement of Alb, especially the reduced Alb, could have a beneficial therapeutic effect on IBD. Using a mouse model of colitis induced by DSS, we tested this hypothesis. Here we present our results demonstrating that reductively modified Alb (r-Alb) effectively attenuates DSS colitis in mice through the –SH group-mediated antioxidative actions.

## 2 Material and methods

### 2.1 Materials

Bovine serum albumin (BSA, Faction V) was obtained from Iwai Chemical Company (Tokyo, Japan). Dimedone was purchased from Tokyo Chemical Industry (Tokyo, Japan), Anti-cysteine sulfenic acid antibody was from Millipore (MA, USA). HRP-conjugated anti-rabbit or mouse IgG, anti-β-actin and anti-p-P38 antibodies were purchased from Cell Signaling, Inc. (Beverly, MA). Anti-E-Cadherin and anti-ZO-1 antibodies were bought from Santa Cruz Biotechnology (Santa Cruz, CA). Alexa 680 Fluor C2 maleimide was from Thermo Scientific (Rockford, IL). Sodium dextran sulfate 5000 (DSS) was from Wako Pure Chemical Corporation (Japan). Maleimide and all other chemicals were from Sigma-Aldrich (St. Louis, MO).

### 2.2 Mice

Both male and female 10–12-week-old C57BL6/J mice were used. Mice could access food and water freely and were housed in quiet rooms with a 12-h light-dark cycle. The animal study was approved by the Animal Care and Use Committee of Yamanashi University and carried out according to the guideline and rules for animal experimentation.

### 2.3 Induction of colitis

Mice colitis was induced by exposing mice to the 3% DSS drinking water for seven days. Mice were treated with different forms of Alb (1 g/kg) or the same volume of control saline (250 μl) once a day with oral gavage or intropeneal injection (i.p.) since the start of DSS administration. Bodyweight (BW) was recorded daily. Feces were collected at various time intervals and stored at 80°C before used for the detection of oxidative status. On day 7, mice were anesthetized for whole blood collection. Afterward, the colon was removed, measured for its length, and stored at 80°C for subsequent analysis.

### 2.4 Sample collection and treatment

Feces at the weight of 100 mg was dissolved in 500 μl PBS containing 1% of a cocktail of proteinase inhibitors (Nacalai Tesque, Kyoto, Japan). After Intense agitation at room temperature in 1.5 ml tubes using a Vortex Genie 2 mixer, the samples were centrifuged at 14,000 g for 15 min. The supernatants were collected, assayed for protein concentration, aliquoted, and stored at 80°C for further experiments. Colon tissues were minced and lysed in RIPA containing 1% protein inhibitor. After sonification and centrifugation, the supernatants were collected and assayed for protein concentration. Blood was collected from the orbital sinus and jugular vein from the sufficiently anesthetized mice. The collected blood samples were placed at room temperature (RT) for 1 h for blood clotting, and the serum was separated by centrifugation.

### 2.5 Western blot analysis

Cellular lysates were prepared using 1 × SDS lysis buffer (62.5 mM Tris-HCl, 2% SDS, 10% glycerol), and tissue samples were homogenized in RIPA lysis buffer together with a freshly added proteinase inhibitor cocktail. After measurement of protein concentration using the Micro BCA Protein Assay Kit (Thermo Fisher Scientific, Waltham, MA), an equal amount of proteins was separated by 10% SDS-PAGE, transferred to PVDF membranes with wet-blotting apparatus. After blocking with 5% skimmed milk or 3% BSA in 0.1% Tween-20 PBS solution (TPBS) for at least 1 h, the membrane was incubated overnight with the primary antibody at appropriate dilution. After washing with TPBS, the blots were incubated with peroxidase-conjugated secondary antibody for 1 h and detected for the bands of the target proteins using the enhanced chemiluminescence system (Nacalai Tesque, Kyoto, Japan). The chemiluminescent signal is captured with a Fujifilm image LAS-1000 analyzer (Fujifilm, Tokyo, Japan), and the intensity of the bands was quantified with the NIH Image J software (http://rsb.info.nih.gov/ij). β-actin was used as an internal control to ensure equal loading of sample protein.

### 2.6 Modification of Alb with reductive chemical DTT

BSA at the concentration of 100–120 mg/ml was incubated with 0.1 M DTT in distilled water at the measured pH of 5.5 overnight at 4 °C, followed by dialysis in a dialysis cassette (7000 MWCO, 3–12 ml) (Thermo Fisher Scientific, Waltham, MA) against a large amount of distilled water or saline (0.9% sodium chloride in distilled water) at the measured pH of 6.5 and 6.0, respectively, with 7 changes of dialysis solution. To block the –SH groups with maleimide, DTT-modified Alb was incubated with 0.1 M maleimide overnight at 4 °C, followed by the same dialysis procedure as above. The thoroughly dialyzed BSA at the measured pH of 6.0 was collected, assayed for protein concentration, examined for the changes in the amount of –SH groups and stored at −80°C. The amount of –SH was confirmed to be stable for at least 6 months after the modification.

### 2.7 Maleimide-labeling assay

This assay was modified from the previous reports [34]. Proteins treated with or without the indicated stimuli were allowed to react with Alexa Fluor 680C2 maleimide (red fluorescence at the final concentration of 10 μM) at 4 °C for 2 h. After the interaction, the protein samples were either directly applied to the PVDF membrane in a Bio-Rad dot-blot apparatus or subjected to SDS-PAGE separation. The signal of fluorescent maleimide in the membranes was captured with a Fujifilm image LAS-1000 analyzer (Fujifilm, Tokyo, Japan) and quantified with ImageJ software. EZ blue staining was performed to confirm the equal loading of proteins.

### 2.8 Detection of thiol state

The number of the reductively modified cysteine residues in Alb was determined using The -SulfoBiotics- Protein Redox State Monitoring Kit following the manufactory's instructions (Catalog #SB11; Dojindo Molecular Technologies Inc., Rockville, MD, USA). Briefly, Alb was reacted with a 15-kDa Protein-SHifter and subjected to non-reducing SDS-PAGE, followed by silver staining using The Pierce Silver Stain Kit. The number of the reduced cysteine residues was determined based on the molecular mass shift of Alb bands after electrophoresis.

### 2.9 Dot-blot

An equal amount of proteins from colon lysates or feces were incubated with Alexa Fluor 680 conjugated C2 maleimide (5 μM, final concentration) and kept for 2 h at 4 °C with occasional gentle mixing. Samples were loaded on PVDF membranes. After thoroughly washing with PBS, the fluorescent signal in the membrane were captured and quantified with the NIH Image J software. EZ blue staining was performed to confirm equal loading of proteins.

### 2.10 Measurement of free SH in albumin using Ellman's reagent

Free thiol concentrations in differently modified Alb were determined using a cysteine standard following the instruction provided by Thermo Scientific with minor modification. Briefly, a set of cysteine standard and samples were dissolved in 0.1 M Tris buffer at pH 8.0 in the presence or absence of 6 M urea for 15 min, followed by incubation with Ellman's reagent (Dojindo Molecular Technologies Inc., Rockville, MD, USA) for an additional 15 min. Absorbance was measured at 412 nm. The concentration of free –SH in samples were calculated based on the standard curves generated from l-cysteine in Tris buffer with or without 6 M urea at pH 8.0 and it was adjusted to albumin concentrations (μmol/g).

### 2.11 Enzyme-linked immunosorbent assay

The collected colon lysates were assayed for IL-1α, β, and TNF-α using an Enzyme-Linked Immunosorbent Assay (ELISA) Kit from Peprotech following the manufacturer's instruction (Rocky Hill, NJ, USA).

### 2.12 Colon histology analysis

Colon histology was performed as we previously described [35]. Briefly, colon tissues were fixed in 10% formalin, embedded in paraffin, sliced into 4-μm sections, and stained with standard hematoxylin and eosin (H&E) procedure.

### 2.13 Detection of sulfenic acid

The protein level of sulfenic acid formation was detected using the method described by Saurin et al. [36]. Briefly, protein samples at the concentration of 1–3 mg/ml were treated with 1 mM dimedone for 20 min at RT, mixed with fivefold non-reducing sample buffer, and subjected to Western blot analysis using an anti-cysteine sulfenic acid antibody.

### 2.14 Cell culture

Caco-2 cells (ATCC, Rockville, MD) were cultured using DMEM/F12 (Gibco-BRL, Gaithersburg, MD, USA) containing 10% FBS and 1% antibiotic and antimycotic solution in a humidified atmosphere of 5% CO_2_/95% air at 37 °C. Human umbilical vein endothelial cells (HUVEC) pooled cells were purchased from Promo Cell (Heidelberg, Germany) and cultured in endothelial cell growth medium 2 (ready-to-use; Takara-bio) for passage and expansion. For experiments, cells were seeded in 12-, 96-well culture plate or inserts until confluence and exposed to different treatments for the indicated times.

### 2.15 Detection of ROS production

The generation of superoxide anion (O_2_^•－^) and ROS was detected by using a commercially available kit from Enzo life sciences (Tokyo, Japan, ENZ-51010) following the manual of the manufacturer, as previously described in our previous reports [35,37]. Briefly, cells in 96-well plates were loaded with O_2_^•^^－^ detection reagent (orange) and oxidative stress detection reagent (green), followed by stimulation with DSS or H_2_O_2_ for 3 h. The fluorescent images were captured using the immunofluorescent microscope (IX71, Olympus, Tokyo, Japan).

### 2.16 Epithelial barrier function

Cell monolayers in 0.33 cm^2^ transwell inserts were exposed to H_2_O_2_ in the presence or absence of different forms of Alb. TER was measured using a Millicell-ERS Electrical Resistance System (Millipore, Bedford, MA) as we have previously reported [38].

### 2.17 Calcein-AM and propidium iodide (PI) staining

Cell viability was determined by the Calcein-AM/PI live/dead staining using an assay kit from Dojindo, Kumamoto, Japan. Briefly, cells were allowed to incubate with a mixture of Calcein-AM (green) and PI (red) solution for 10–20 min and observed under a fluorescent microscope. Calcein-AM positive green cells were considered alive, while PI-positive red cells were considered dead.

### 2.18 Assessment of cell viability with WST reagent

Cells grown in 96-well culture plates were exposed to WST reagent (Dojindo, Kumamoto, Japan) for 60 min. The optical density (OD) was measured with a spectrometer at the wavelength of 450 nm.

### 2.19 Lactate dehydrogenase (LDH) assay

Cell cytotoxicity was quantitatively assessed using a commercial kit following the manufacturer's instruction (LDH Cytotoxicity Detection Kit, TaKaRa). Briefly, cells were seeded into a 96-well plate and grown to 80–90% confluence in DMEM/F12 supplemented with FBS. After stimulation, the supernatant was collected and allowed to react with the same volume of assay buffer at RT for 30 min. The intensity of red color formation was measured using a spectrometer at 490 nm. LDH release was calculated and expressed as the percentage of the total release. The culture media was used as the background control; cell lysate obtained from 2% Triton X-100 was taken as 100% release.

### 2.20 Analysis of hydrogen peroxide-quenching capacity of the modified Alb

The quenching capacity of the modified Alb on H_2_O_2_ was detected using a similar method as described by Anraku et al. [24]. It is based on the principle that the neutralization of H_2_O_2_ by Alb leads to a decreased availability of H_2_O_2_ to activate ROS prober, causing a lower level of fluorescence emission. Briefly, H_2_O_2_ was allowed to react with the differently modified Alb for 30 min. An aliquot of the solution (50 μl) was mixed with the same volume of 1:250 diluted total ROS detection reagent Green (Enzo life sciences, Tokyo, Japan), and incubated for an additional 30 min in the dark at RT, followed by fluorescence measurement at 540/595 nm excitation/emission.

### 2.21 GSH/GSSG assay

For the measurement of reduced glutathione (GSH) or oxidized glutathione (GSSG), GSH/GSSG Quantitative Kit was used following the manufacturer's instructions (Product code: G257; Dojindo Molecular Technologies, Inc.).

### 2.22 Statistical analysis

Values are expressed as mean ± SD. Comparison of two groups was made by Student's t-test. For multiple comparisons with the same control, one-way ANOVA analysis and Post-hoc comparisons were performed. Both analyses were done using Microsoft Excel (Microsoft, Redmond, WA, USA) or Sigmaplot software. P < 0.05 was considered statistically significant.

## 3 Results

### 3.1 Induction of DSS colitis is associated with oxidative stress

DSS colitis was induced by exposing mice to 3%-DSS drinking water (Fig. 1A). The mice developed the typical symptoms of colitis, such as the fecal occult blood (Fig. 1B) and diarrhea, starting from day 3 after DSS administration. A significant loss of BW was observed on day 7 (Fig. 1C). The colon length was shortened, and the thickness was decreased. Signs of colon inflammation and bleeding were observed at necropsy (Fig. 1D). Histopathological analysis and cytokine detection revealed that there existed colon epithelial cell injury and inflammation (described in the next part of the result).

**Fig. 1:**
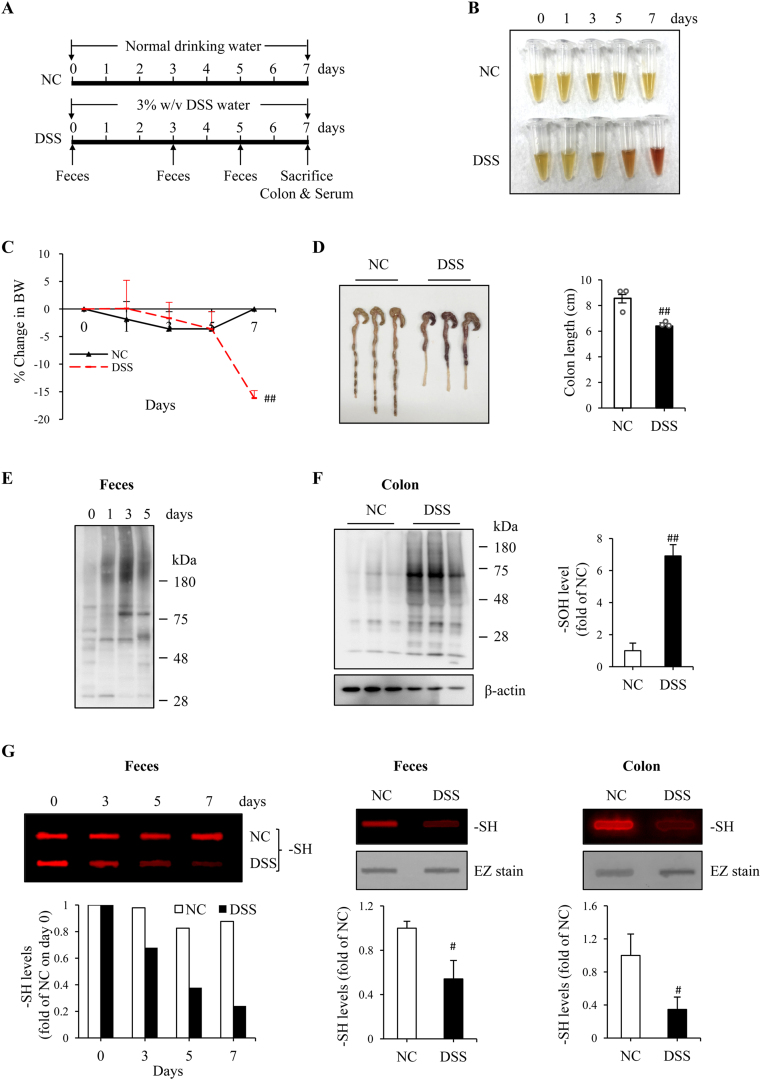
*DSS colitis is associated with oxidative stress in colon tissues.* (A) Schematic diagram to illustrate the experimental design. (B) Stool color in differently treated mice. The feces collected from normal control (NC) and DSS-treated mice were lysed in PBS solution. The color of the supernatants was photographed. Note the obvious color change in the DSS group. (C) The change in mice BW. The BW was daily recorded and expressed as the percentage of BW loss relative to BW on day zero. Data shown are mean ± SD (n = 3; ^##^P < 0.01 vs. NC). (D) The change in colon length. The colon was taken at necropsy on day 7 and photographed (Left panel). The colon length was measured and expressed as centimeter in bar graph (mean ± SD, n = 3; ^##^P < 0.01). (E, F) Detection of sulfenic acid formation in fecal and colon proteins. An equal amount of proteins from feces or colon tissues was treated with dimedone and subjected to Western analysis for sulfenic acid (-SOH) using an anti-cysteine sulfenic acid antibody. β-actin is used as a loading control. The densitometric quantification of the signals in F was shown in bar graph (mean ± SD, n = 3; ^##^P < 0.01). (G) Detection of –SH groups in feces and colon tissues. Proteins extracted from feces and colon tissues were allowed to react with fluorescent maleimide and the level of –SH was detected using dot blot analysis. The densitometric values are presented as fold of control. The result of the time-course changes of –SH in feces was from a single experiment. The data of others are mean ± SD (n = 4; ^#^P < 0.05). (For interpretation of the references to color in this figure legend, the reader is referred to the Web version of this article.)

The development of colitis was associated with oxidative stress in the colon. The level of sulfenic acid formation was progressively elevated in fecal proteins, being detectable as early as day 1 after DSS administration (Fig. 1E). It was also significantly increased in colon tissues (Fig. 1F). The increases were associated with a decreased level of –SH groups in feces and colon, as revealed by fluorescence-based maleimide-labeling assay (Fig. 1G). These observations indicate that the induction of DSS colitis is associated with increased protein oxidation and decreased availability of thiol antioxidants.

### 3.2 Oral administration of r-Alb alleviates mice DSS colitis

To determine whether treatment of colitis with the reduced form of Alb could affect the course of DSS colitis. We first prepared and characterized the reductively modified albumin (r-Alb). Fig. 2A shows that modification of Alb with reductive chemical DTT caused a marked increase in the amount of –SH groups, as revealed by the increased binding of Alb to the fluorescent maleimide. To establish the role of –SH groups in the antioxidative effects, we also took advantage of the thiol-binding property of maleimide and blocked –SH groups in r-Alb. The treatment with normal maleimide completely abolished the subsequent binding of r-Alb to the fluorescent maleimide, indicating a total blockade of –SH groups.

**Fig. 2:**
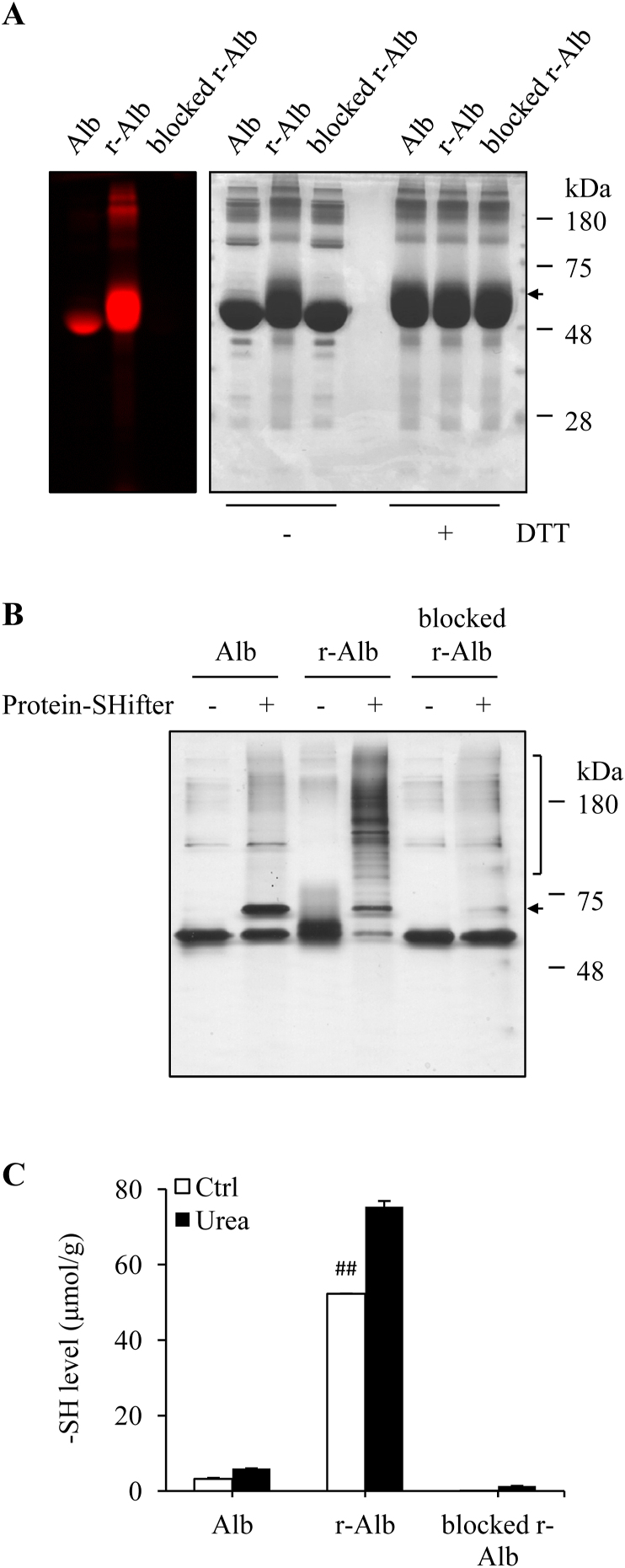
*Treatment of Alb with DTT results in an increased number of -SH groups.* (A) Effect of DTT treatment on the number of –SH groups in Alb. BSA at the concentration of 100 mg/ml was treated with or without 0.1 M DTT overnight at 4 °C, followed by repeated dialysis to remove remaining DTT and other small MW metabolites. The modified Alb was subjected to fluorescence-labeled maleimide assay for –SH groups. Note the increased fluorescent intensity in r-Alb and its abolishment after blockade with maleimide (left image). EZ blue staining of the gel was performed to confirm the equal loading of the samples (right image). Because r-Alb exhibited a more diffused band after running SDS-PAGE, which influenced the judgment, all the samples were also treated with DTT to show the equal loading. (B) Determination of the numbers of cysteines to be reductively modified by DTT with Protein Redox State Monitoring Assay. Differently modified Alb was reacted with Protein-Shifter and subjected to SDS-PAGE separation, followed by silver staining of proteins. Note that only one shifted band was detected in normal Alb (arrow), whereas more than 10 additional bands were found in r-Alb (square bracket) and its disappearance after blockade with maleimide. (C) Determination of –SH group using Ellman's reagent. Differently modified Alb was assayed for –SH concentration using Ellman's reagent and the concentration was expressed as μmol/g (n = 4; ^##^P < 0.01 vs. Alb). (For interpretation of the references to color in this figure legend, the reader is referred to the Web version of this article.)

Structurally, Alb has a total of 35 cysteine residues, forming 17 intra-chain disulfide bonds and leaving Cys34 as the only reactive –SH group. To determine the number of cysteines to be reductively modified by DTT, we detected the redox state of the modified Alb using Protein Redox State Monitoring Kit. This assay kit is based on the principle that the binding of a 15-kDa Protein-SHifter to the –SH group of the target protein results in a molecular mass shift in SDS-gel [39]. Fig. 2B shows that in normal Alb, only one additional band at the position of 15-kDa upward of the original Alb was detected, and the intensity of the band was almost the same as the original one, revealing that the half of the Alb used in this investigation was reduced Alb with one reactive SH group. In stark contrast to normal Alb, more than 10 additional bands, ranging from 80 kDa to 250 kDa, were detected in r-Alb, accompanied by a marked decreased band of Alb at the original position. As expected, these changes were disappeared in maleimide-blocked r-Alb, indicative of an entire blockade of –SH groups.

Consistent with the above results, assay of –SH groups using Ellman's reagent also revealed the level of –SH groups in DTT-treated Alb (r-Alb) was significantly higher than control under both native and urea-denatured conditions. It was increased up to 16 folds of control after reductive modification and could be completely blocked by maleimide (Fig. 2C). Collectively, these results suggest that the reductive treatment markedly increased free reactive –SH groups in Alb.

Using the differently modified Alb, we then investigated the possible therapeutic effects on DSS-induced mice colitis. Mice were divided into four groups: normal control (NC), DSS control, DSS mice treated with normal Alb, and DSS mice treated with r-Alb. For induction of DSS colitis, mice were allowed to access 3% DSS with drinking water freely for 7 days. Alb was administrated via gavage once daily at the dosage of 1 g per kilogram BW, starting from DSS administration. Control mice received the same volume of saline. BW was monitored daily. Feces were collected on day 0, 3, 5 and 7. Colon length, histopathologic and inflammatory changes were examined. Fig. 3 shows that treatment of mice with r-Alb significantly improved disease activity. The symptoms of the fecal occult blood and diarrhea were disappeared. The black stool, a sign of intestinal bleeding, occurred in DSS colitis starting from day 3–5, was not observed in the group treated with the r-Alb (Fig. 3A). The loss of BW, the shorten length of the colon, and the signs of inflammation and bleeding in DSS group were also attenuated by r-Alb (Fig. 3B and C).

**Fig. 3:**
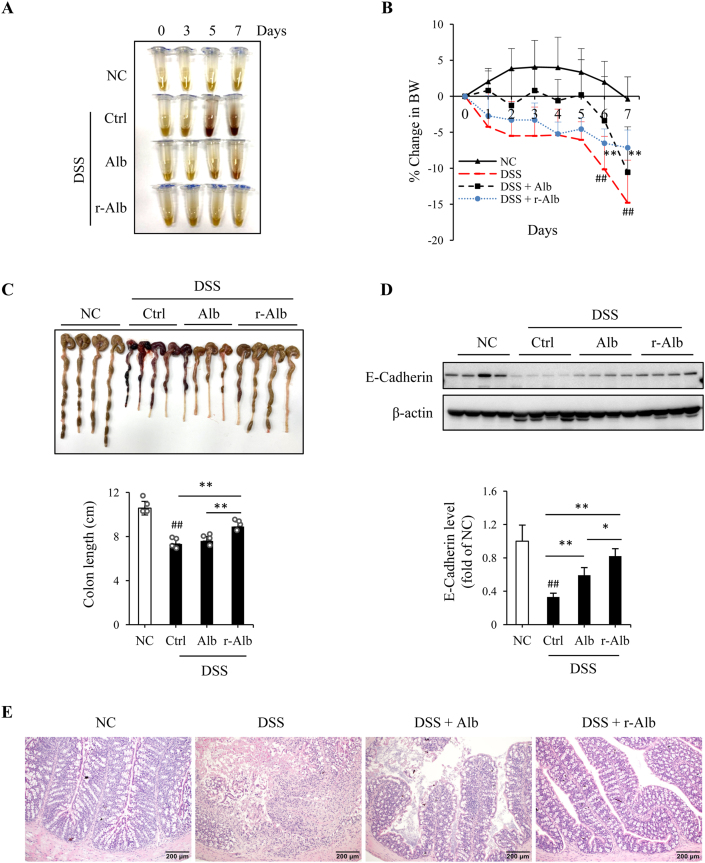
*Oral administration of Alb prevents the development of DSS colitis.* (A) Effect of Alb treatment on stool color. The color of the lysed stool was photographed. (B) Effect of Alb on the change of mice BW. The BW was daily recorded and expressed as the percentage of BW loss relative to day zero. Data shown are mean ± SD (n = 5; ^##^P < 0.01 vs. NC; **P < 0.01 vs. DSS). (C) Effect of Alb treatment on the colon length. The colon taken from necropsy on day 7 was photographed and measured for its length. Data shown in bar graph are mean ± SD (n = 4; ^##^P < 0.01 vs. NC; **P < 0.01). (D) Effect of Alb treatment on the level of E-Cadherin. Protein extracted from colon tissues was subjected to Western blot analysis for E-Cadherin. The densitometric value is presented in bar graph. Data are expressed as fold of NC (mean ± SD; n = 4; ^##^P < 0.01 vs. NC; *P < 0.05, **P < 0.01). (E) Effect of Alb treatment on the histopathological changes in the colon. Representative images of HE staining of colon tissues of DSS mice on day 7 are shown. Note that DSS-induced colon tissue had destruction of the epithelial architecture, including the loss of crypts and epithelial integrity, submucosal edema, and intense infiltration of inflammatory cells. These changes were partially alleviated in the mice treated with normal Alb and completely abolished by r-Alb. (For interpretation of the references to color in this figure legend, the reader is referred to the Web version of this article.)

Further analysis of colon tissues revealed that DSS administration caused a loss of adherens junction protein E-cadherin, which was largely prevented by r-Alb treatment (Fig. 3D). Histological analysis revealed that the pathological changes, such as immune cell infiltrates, loss of goblet cells, crypts, and villi, epithelial erosions, and ulcers in the DSS group were all disappeared in r-Alb -treated mice (Fig. 3E). Intriguingly, unmodified Alb also alleviated some of the pathological changes in DSS colitis, but it was far less effective than r-Alb.

This therapeutic effect of r-Alb was associated with a noticeable improvement in the inflammatory and oxidative state (Fig. 4). The increased production of inflammatory mediator IL-1α, IL-1β, and TNF-α in colon was inhibited up to normal level (Fig. 4A). Similarly, the formation of sulfenic acid in colon and feces was also strongly suppressed (Fig. 4B and C). Fascinatingly, r-Alb treatment also reduced sulfenic acids in blood proteins (Fig. 4D). It markedly inhibited DSS-induced sulfenic formation in several serum proteins (arrow-marked positions). In contrast to the impressive therapeutic effects of r-Alb, normal Alb only partially improved inflammatory and redox status, as well as disease activity. Collectively, the administration of r-Alb improves disease activity and rebalances the body redox state.

**Fig. 4:**
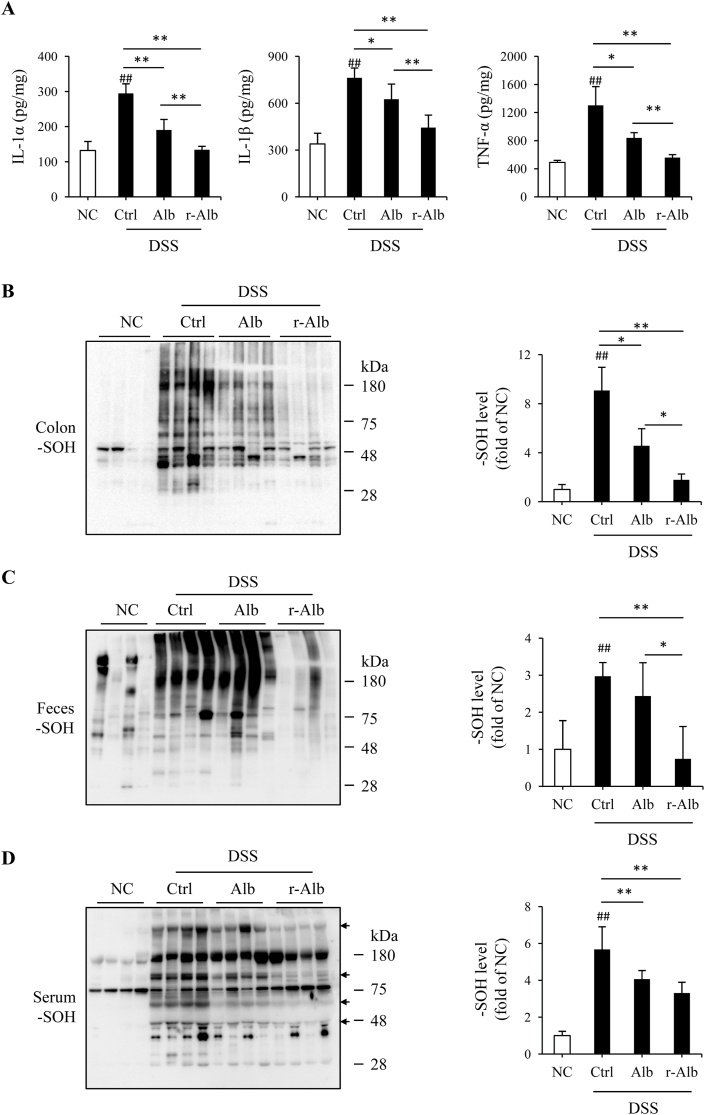
*Alb treatment improves inflammatory and redox state in DSS colitis.* (A) Effect of Alb treatment on inflammatory cytokines in the colon. Protein extracted from colon tissues on day 7 after DSS administration was measured for the level of IL-α, β, and TNF-α with ELISA. Data shown are mean ± SD (n = 4; ^##^P < 0.01 vs. NC; *P < 0.05, **P < 0.01). (B–D) Effect of Alb treatment on sulfenic acid formation in colon, feces, and serum. Proteins obtained from colon tissues, feces, and serum were treated with dimedone and subjected to Western blot analysis for sulfenic acid formation. The densitometric quantification of the signals in the blots was performed and the data are presented in the bar graph at the right side of the blot (mean ± SD; n = 4; ^##^P < 0.01 vs. NC; *P < 0.05, **P < 0.01).

### 3.3 Intraperitoneal injection of the r-Alb alleviates DSS-induced colitis

**Fig. 5:**
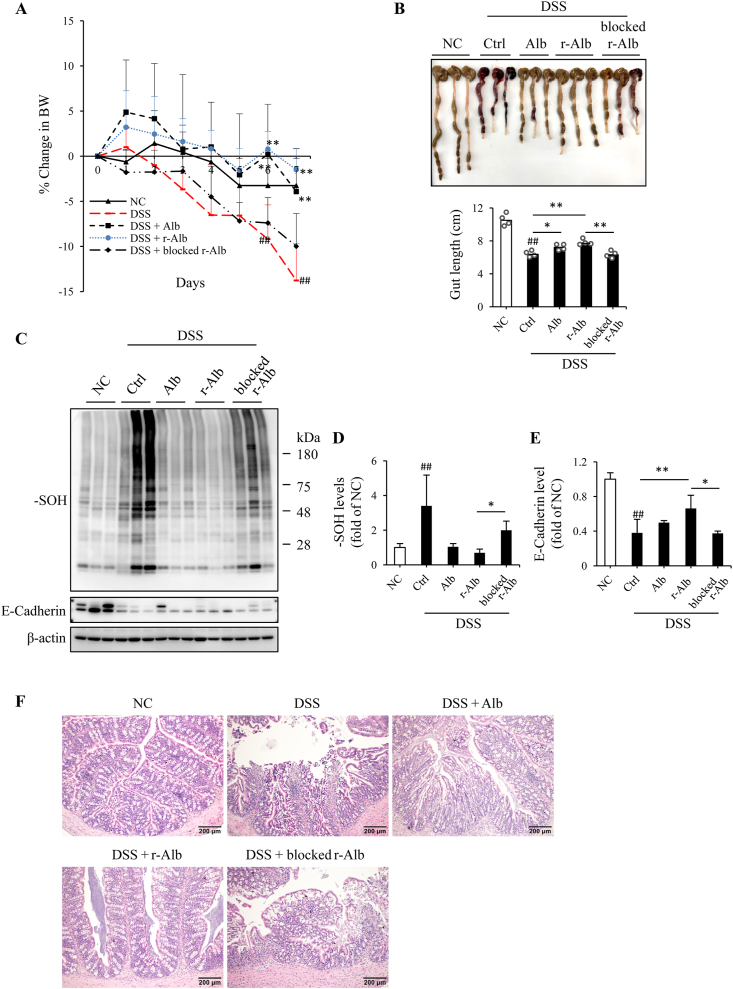
*Intraperitoneal injection of Alb attenuates the disease activity of DSS colitis.* (A) Effect of Alb treatment on BW. Mice were allowed to access 3% DSS with drinking water freely for 7 days. Alb-treated mice received 1 g Alb per kilogram BW via intraperitoneal injection once daily. Control mice received the same volume of saline. The BW was daily recorded and expressed as the percentage of weight loss against the BW on day zero. Data shown are mean ± SD (n = 5; ^##^P < 0.01 vs. NC; **P < 0.01 vs. DSS). (B) Effect of Alb treatment on colon length. The colon on day 7 was photographed. The colon length was measured and expressed as centimeter in bar graph (mean ± SD; n = 3; ^##^P < 0.01 vs. NC; *P < 0.05, **P < 0.01). (C–E) Effect of Alb treatment on colon sulfenic acid formation and adherens junction protein. Protein extracted from colon tissues of differently treated mice was subjected to Western blot analysis for sulfenic acid and E-Cadherin. The densitometric quantitation of the bands in (C) is shown in D and E. Data are expressed as fold of NC (mean ± SD; n = 3; ^##^P < 0.01 vs. NC; *P < 0.05, **P < 0.01). (F) Effect of Alb treatment on histopathological changes. Representative images of HE staining of the colon are shown. Note that DSS-induced colon tissue had partial destruction of the epithelial architecture, including the loss of crypts and epithelial integrity, submucosal edema, and inflammatory cell infiltration. These changes were markedly inhibited in the mice treated with r-Alb.

As a biological product, Alb is frequently used in clinical practice via intravenous injection. We, therefore, asked whether administration of Alb through other route, such as i.p., could have the same therapeutic effect. Fig. 5 shows that daily i.p. injection of r-Alb also alleviated DSS colitis, as indicated by the improved BW, colon length, the level of adherens junction protein, protein oxidation and histopathological changes. To establish the role of –SH groups in the therapeutic effects, we included an additional group in which the mice were treated with the maleimide-blocked r-Alb, and found that blockade of –SH groups in r-Alb largely abolished the observed therapeutic effects.

### 3.4 R-Alb prevents H_2_O_2_-induced oxidative cell injury through its –SH groups

DSS-induced colitis is associated with increased ROS production [40]. Using a cultured human epithelial colorectal adenocarcinoma cell Caco-2, we confirmed the previous report. Exposure of Caco-2 cells to DSS caused an elevation in total ROS and superoxide (Supplementary Fig. 1A). The intensity of cellular fluorescence, which is proportionally correlated with intracellular ROS (green) and superoxide (red), was increased. Furthermore, in support of a mediating role of oxidative stress in the effect of DSS, supplement of cells with thiol-antioxidant GSH or GSH precursor NAC (data not shown) significantly prevented DSS-induced cell injury. The number of PI-positive red cells, the amount of extracellular LDH as well as the loss of cell viability were all significantly inhibited (Supplementary Fig. 1, B-D).

**Fig. 6:**
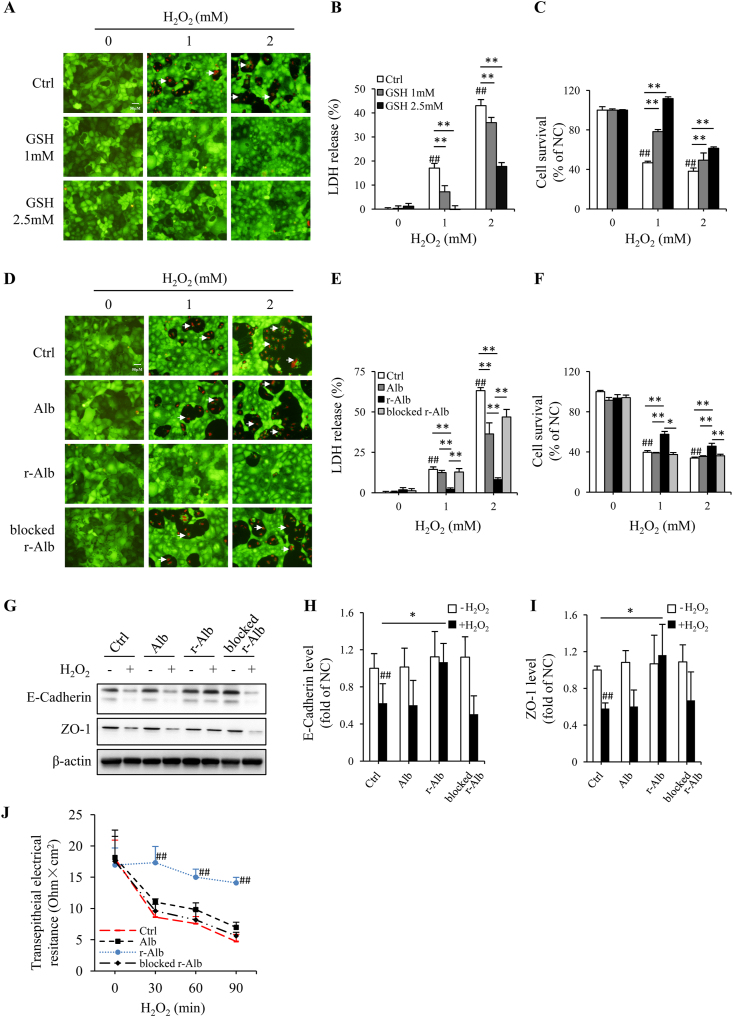
*Thiol antioxidant GSH and r-Alb prevent* H_2_O_2_*-induced oxidative cell injury in colon epithelial cell line Caco-2.* (A–F) Induction of oxidative cell injury by H_2_O_2_ and its prevention by thiol antioxidant GSH and r-Alb. Cells were exposed to the indicated concentrations of H_2_O_2_ in the presence or absence of the indicated concentrations of GSH or 5 mg/ml modified Alb for 6 h. The cell viability was determined using Calcein AM/PI staining (A, D), LDH release (B, E), and formazan formation (C, F). The PI-positive dead cells in (A, D) have been arrow-marked. The data in B, C, E and F are mean ± SD (n = 4; ^##^P < 0.01 vs. NC; *P < 0.05, **P < 0.01). (G–I) Effect of differently modified Alb on H_2_O_2_-induced changes in E-Cadherin and ZO-1. Cells were exposed to 600 μM H_2_O_2_ in the presence or absence of 2.5 mg/ml modified Alb for 3 h. The cellular proteins were extracted and subjected to Western blot analysis for E-cadherin and ZO-1 (G). The densitometric analysis of the blot in G is shown in H and I. The results are expressed as the percentage of change relative to untreated control. Data shown are mean ± SD from four separate experiments (n = 4; ^##^P < 0.01 vs. NC; *P < 0.05). (J) Time-course effect of differently modified Alb on H_2_O_2_-induced disruption of barrier function. Confluent Caco-2 cells were exposed to 2 mM H_2_O_2_ in the presence or absence of differently modified Alb (5 mg/ml) for the indicated time intervals and subjected to TER measurement. TER was expressed as the ratio of voltage to current normalized by the area of the monolayer. Data shown are mean ± SD (n = 4; ^##^P < 0.01 vs. respective control).

Given that ROS, especially H_2_O_2_, plays a central role in DSS colitis [4,[41], [42], [43], [44]], we, therefore, asked whether the supplement of cells with different forms of Alb could prevent H_2_O_2_-induced cell injury in Caco-2 cells. As shown in Fig. 6A, similar to the small thiol antioxidant GSH, r-Alb potently inhibited H_2_O_2_-induced cell injury, as indicated by the reduced number of PI-positive dead cells (red; Fig. 6, A and D), the decreased level of extracellularly released LDH (Fig. 6, B and E), as well as the increased cell viability (Fig. 6, C and F).

Because disruption of adherens/tight junction is an early and reversible marker of epithelial cell injury, which plays a pivotal role in the onset of DSS colitis [8], we also examined the effect of Alb on H_2_O_2_-induced loss of barrier function. The results showed that r-Alb prevented the loss of E-cadherin and ZO-1, as well as the early dysfunction of transepithelial electrical resistance (TER) induced by H_2_O_2_ in a –SH group-dependent way (Fig. 6, G-J).

### 3.5 Alb detoxifies H_2_O_2_ through –SH group-dependent mechanism

**Fig. 7:**
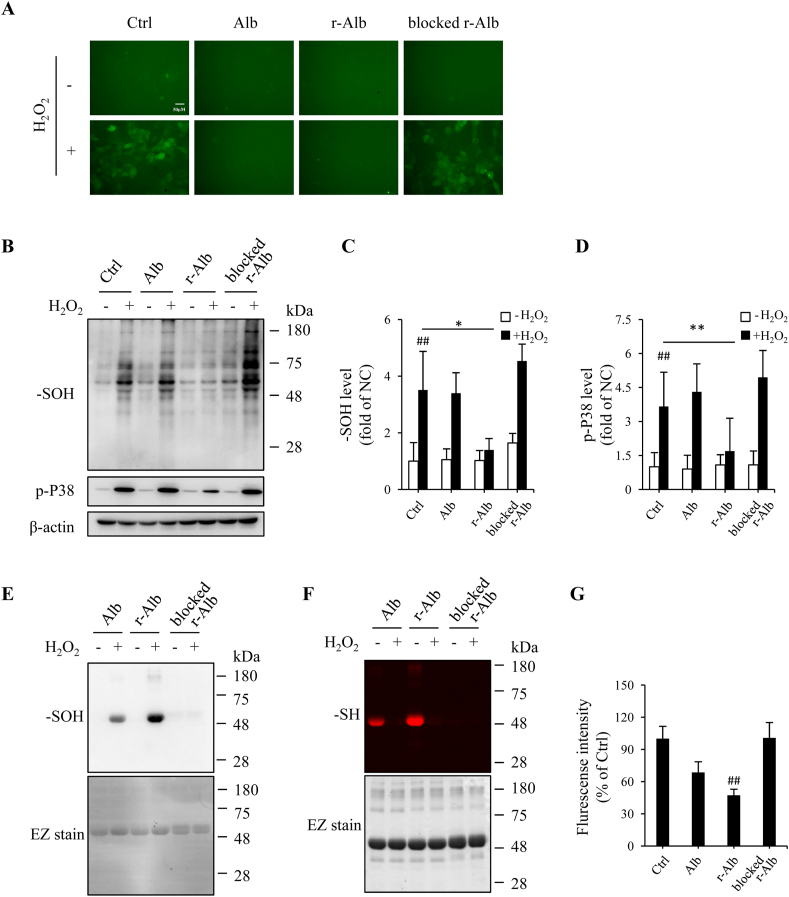
*R-Alb interferes with H*_*2*_*O*_*2*_*-initiated intracellular ROS level and cell protein oxidation.* (A) Effect of differently modified Alb on H_2_O_2_-induced intracellular level of total ROS. Caco-2 cells were exposed to 300 μM H_2_O_2_ that had been pre-incubated with 2.5 mg/ml modified Alb for 30 min before the addition. After 3 h, the intracellular level of total ROS (green) was detected. Note the obvious reduction in the fluorescent intensity in the group pretreated with r-Alb. (B–D) Effect of differently modified Alb on H_2_O_2_-induced formation of sulfenic acids in cellular proteins and activation of P38. Cells were exposed to 600 μM H_2_O_2_ in the presence or absence of 2.5 mg/ml modified Alb for 3 h. The cellular proteins were extracted and subjected to Western blot analysis for sulfenic acid (B). The densitometric analysis of the blot in B is shown in C and D. The results are expressed as the percentage of change relative to untreated control. Data shown are mean ± SD from four separate experiments (n = 4; ^##^P < 0.01 vs. NC; *p < 0.05; **P < 0.01). (E, F) Sulfhydryl group-meditated interaction between H_2_O_2_ and Alb. One mg/ml modified Alb were incubated with 1 mM H_2_O_2_ for 1 h at RT. Afterward, an aliquot of the reaction solution was incubated with 1 mM dimedone to detect sulfenic acids (E) or 10 μM fluorescent maleimide for determination of –SH groups (F). The same membrane and gel were stained with EZ blue to confirm the equal loading of proteins in each lane. (G) Treatment of H_2_O_2_ with differently modified Alb on the H_2_O_2_ level. Three hundred micromolar H_2_O_2_ were incubated with 2.5 mg/ml Alb for 30 min at RT. Afterward, ROS detection prober was added at the dilution of 1:500 for an additional 30 min. The fluorescent intensity was detected at the wavelength of 490/525 nm. The data are expressed as relative optical density against H_2_O_2_ alone (mean ± SD; n = 4; ^##^P < 0.01 vs. Ctrl). (For interpretation of the references to color in this figure legend, the reader is referred to the Web version of this article.)

The protective action of r-Alb on H_2_O_2_-induced epithelial injury prompted us to investigate the potential mechanisms involved. It is known that Alb is susceptible to oxidative modification [[45], [46], [47]]. We speculated that the reaction of –SH groups of Alb with H_2_O_2_ might spare cells from H_2_O_2_ toxicity. To test this hypothesis, we determined the intracellular ROS and ROS-mediated cellular protein oxidation after exposing the cells to exogenous H_2_O_2_ with or without the differently modified Alb. As expected, the addition of H_2_O_2_ into Caco-2 cells, which had been preloaded with the ROS indicator, led to an increased fluorescent signal in the cells, suggesting an elevation in intracellular ROS (Fig. 7A). In the presence of r-Alb, however, the elevation was completely abolished. Consistently, r-Alb also abolished the H_2_O_2_-induced sulfenic acid formation in cellular proteins and activation of P38 (Fig. 7B–D). Apart from colon epithelial cells, r-Alb also potently inhibited H_2_O_2_-induced changes in human umbilical vein endothelial cells (Supplementary Figure 2) and DSS-induced changes in Coca-2 cells (data not shown).

Further analysis confirmed that r-Alb was more susceptible to H_2_O_2_-induced protein oxidation. A higher level of sulfenic acids was detected in r-Alb than normal Alb (Fig. 7E). The discrepancy was due to their difference in the amount of –SH groups. Blockade of –SH groups with maleimide abolished sulfenic acid formation. In further support of this notion, the formation of sulfenic acids was associated with a loss of –SH groups (Fig. 7F).

To further confirm the detoxification of H_2_O_2_, we detected H_2_O_2_ concentration. Fig. 7G shows that the treatment of H_2_O_2_ with r-Alb significantly lowered its ability to activate the fluorescence emission from H_2_O_2_-reactive ROS probe.

### 3.6 Alb and GSH/GSSG interaction reciprocally regulate the activity of –SH groups

**Fig. 8:**
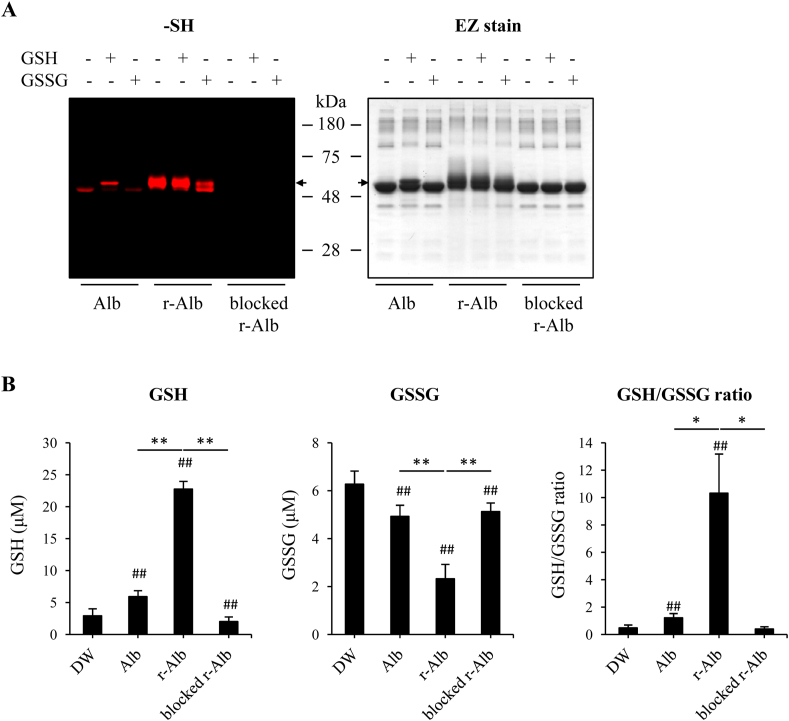
*Alb and GSH/GSSG interaction reciprocally regulate the activity of -SH groups.* (A) Effect of GSH and GSSG on the number of –SH groups in the differently modified Alb. Differently modified Alb was allowed to react with 2 mM GSH or GSSG overnight at 4 °C. After removing the unreacted GSH and GSSG, the equal number of treated samples was assayed for SH groups using the fluorescent maleimide-labeling assay. The samples were separated with 10% SDS-PAGE and detected for the fluorescent signal. The equal loading of proteins was confirmed by staining the gel with EZ blue. Note the changes in the fluorescent intensity of bands and the shift of bands after treatment with GSH or GSSG. (B) Influence of the modified Alb on GSH and GSSG ratio. A mixture of GSH and GSSG at the estimated concention of 5 μM was incubated with different forms of Alb (0.1 mg/ml in 100 μl distilled water) for 60 min and assayed for GSH and GSSG concentrations. Data shown are mean ± SD (n = 4; ^##^P < 0.01 vs. DW; *P < 0.05, **P < 0.01). (For interpretation of the references to color in this figure legend, the reader is referred to the Web version of this article.)

Because the ratio of GSH and GSSG has been extensively used as an indicator for evaluation of in vivo redox state [18,48], we tested whether there might exist a reciprocal regulation between GSH/GSSH and Alb through thiol-disulfide exchange. To this end, we have incubated different forms of Alb with GSH or GSSG and detected the changes in –SH groups. Fig. 8A shows that incubation with GSH caused an increased number of –SH groups in normal Alb, whereas treatment with the pro-oxidant GSSG caused a decrease in –SH groups in the normal Alb and r-Alb. Gel staining revealed that treatment of Alb with reductive chemical GSH or DTT caused an upward shift of Alb band, which was disappeared after treatment with GSSG, suggesting that GSH and GSSG modified Alb in a opposite way.

We also looked at the influence of Alb on GSH/GSSG ratio. Fig. 8B shows that the addition of r-Alb at the concentration of 0.1 mg/ml into the mixture of GSH/GSSG caused an increase in GSH activity and a decreased in GSSG activity. Consequently, it drastically elevated the ratio of GSH/GSSG. Treatment of r-Alb with maleimide abolished the observed effects. It is worth mentioning that the assay kit used for measurement of GSH/GSSG is based on the reaction of –SH with DTNB reagent. The –SH in r-Alb was also be detected. Therefore, the activity we detected should be GSH-like activity, including –SH from both GSH and Alb. Taken together, these results suggest an existence of a thiol-mediated reciprocal regulatory loop among GSH, GSSG, and Alb, which may contribute to the maintenance of redox homeostasis in vivo.

## 4 Discussion

**Fig. 9:**
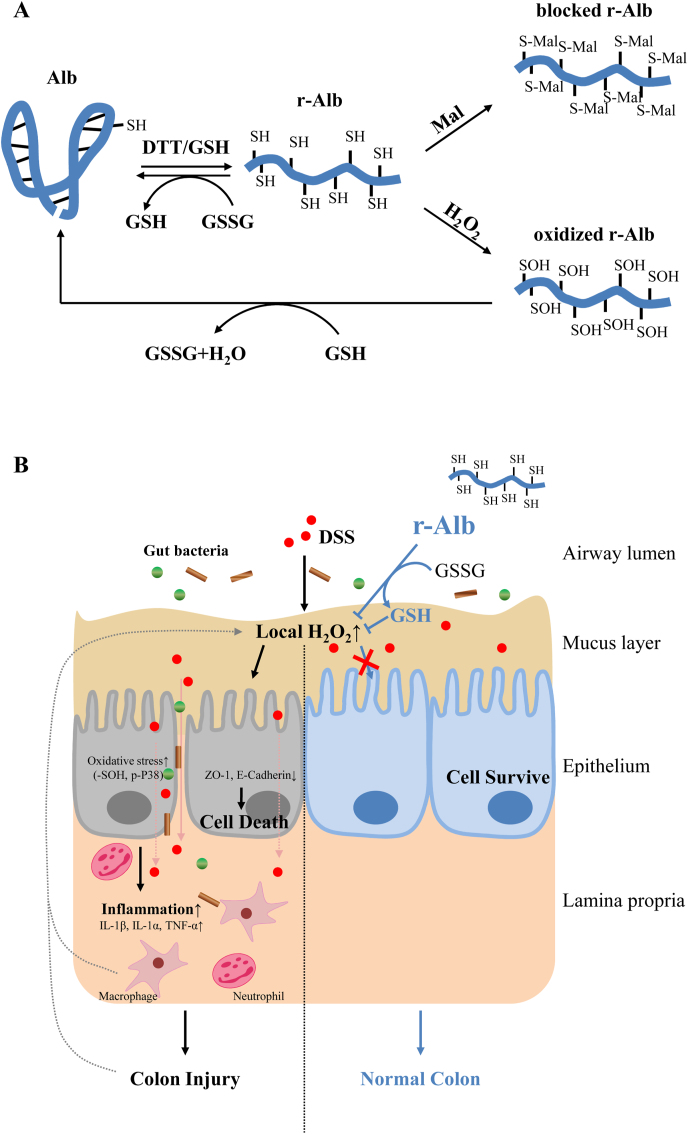
*Schematic depiction of the modified Alb and its role as well as mechanisms in the treatment of DSS colitis.* (A) Reductively modified Alb and its –SH group-mediated interaction with other redox molecules. DTT treatment dramatically increases the number of –SH groups through maintaining Cys34 at the reduced state and cleaving the intra-chain disulfide bonds. The increased –SH groups in Alb react with H_2_O_2_, causing sulfenic acid formation (-SOH), which can be further detoxified in the presence of additional –SH groups from Alb or other small thiol antioxidant GSH. The –SH group-mediated interaction between Alb and GSH/GSSG contributes to maintaining systemic redox homeostasis. (B) Therapeutic effects of r-Alb on DSS colitis. DSS induces ROS production in intestinal epithelial cells, which results in the disassembly of adherens and tight junction, leading to bacterial invasion, recruitment of inflammatory cells, local inflammatory reactions, ROS releasing, and cell injury. R-Alb detoxifies the major oxidant H_2_O_2_ and subsequently attenuates ROS-induced inflammation and cell injury.

In this study, we demonstrated that the treatment of DSS colitis with r-Alb effectively attenuated the disease activities. This effect of r-Alb was associated with a systemic improvement in the oxidative state through mechanisms involving its –SH group-mediated interaction with oxidants. The schematic depiction of our findings and the potential mechanisms are shown in Fig. 9.

DSS colitis is a well-used model for elucidating the mechanisms of the inflammatory bowel diseases, which resembles UC in the intestinal symptoms and inflammatory pathological changes. Using this model, we demonstrated that the administration of r-Alb potently prevented the onset and progression of the disease. In patients with colitis, the amount of plasma Alb is decreased because of the chronic inflammation in the intestinal tract, and the antioxidative capacity of Alb is also weakened due to the oxidative modification [22,[31], [32], [33]]. As a bioproduct, Alb is used in many clinical situations. It is conceivable that the supplement of r-Alb could be a practical and promising way to treat colitis.

The therapeutic actions of r-Alb on colitis could be ascribed to its antioxidative activity. DSS is a polyanionic sulfated derivative, which induces oxidative stress through direct reaction with many biomolecules. DSS colitis is associated with massive oxidative stress, as indicated by the increased level of superoxide and H_2_O_2_, as well as the decreased level of antioxidants [4,5,49]. Strategies in the suppression of ROS production and enhancement of antioxidative machinery are useful in treating colitis in both experimental animal models and clinical patients [[11], [12], [13]]. In consistence with these previous reports, we confirmed that DSS induced ROS generation in cultured colon epithelial cells (Supplementary Figure 1). Furthermore, using thiol oxidation as a marker, we demonstrated the presence of systemic oxidative stress in DSS-administrated mice. The therapeutic effects of r-Alb were strongly associated with a better oxidative status in local tissues and blood, implying that the effect of Alb was through rebalancing the whole-body redox state.

The question naturally occurs as to how Alb regulated body redox balance. ROS, such as H_2_O_2_, superoxide, nitric oxide, peroxynitrite, and hypochlorous acid has been reported to be increased in a variety of diseases. It underlies many pathological changes and correlates with inflammatory activities [4,5,49]. Among ROS, H_2_O_2_ is particularly important. H_2_O_2_ is the product of the SOD-catalyzed dismutation of superoxide, the main ROS formed at most sites of production [49,50]. It is the precursor of several toxic radical species, such as peroxyl radical, hydroxyl radical, and superoxide. Moreover, unlike other radicals, H_2_O_2_ can pass over cell membranes to transmit free radical-induced damage across cell compartments and between cells. A large amount of H_2_O_2_ released by inflammatory cells contributes to bacterial killing, and it also causes cell and tissue injury. In DSS colitis and UC patients, increased H_2_O_2_ in local tissues was reported [4,51]. H_2_O_2_ solution used for the endoscope or enema has been reported to induce colitis [52,53]. Treatment of colitis with catalase, the main enzyme responsible for H_2_O_2_ decomposition, attenuated the colitis [41,54,55]. Consistently, we found that the level of sulfenic acid, the main product of the reaction between a protein cysteine thiol and H_2_O_2_ [56], was significantly increased. Intriguingly, the elevation was not limited at the colon, but also in blood, suggesting an increased H_2_O_2_ in extracellular fluids. Because the amount of catalase in the blood is low, and it was reported to be suppressed in DSS colitis and in patients with Crohn's disease [12,21]. It is conceivable, under such conditions, that detoxification of H_2_O_2_ in serum is mainly carried out by thiol antioxidants, in which Alb should play an important role. Detoxification of H_2_O_2_ and its hydroxyl radical products by Alb has been described in several papers [25,45,47,57]. In this study, we provided evidence showing that r-Alb prevented H_2_O_2_-induced oxidative responses and cell injury in both in vitro and in vivo models. The potent protective effects of r-Alb could be explained by several inter-dependent mechanisms. First, the thiol resident in Alb is highly susceptible to H_2_O_2_-induced thiol oxidation [26,28,58,59]. The markedly increased number of –SH groups made Alb even more likely to be targeted by H_2_O_2,_ thus sparing the other critical cellular proteins from oxidative damage. Second, the increased –SH groups in Alb rendered it even more powerful in scavenging H_2_O_2_. Third, we demonstrated that there existed a reciprocally interactive loop between Alb and the major small thiol antioxidant GSH through –SH exchange. As an integral part of thiol antioxidative system, Alb may exert its antioxidative actions more efficiently through the interaction and coordination with other thiol antioxidants. The interaction could also be the mechanism by which Alb participates in the maintenance of the systemic redox balance. Lastly, Alb also has –SH group-independent antioxidative actions, which could also contribute to the observed protective effects.

The disruption of adherens and tight junctions is a hall marker of epithelial cell injury, which plays a crucial role in the pathogenesis of many diseases, including colitis. Normally, the epithelial cell adherens and tight junction acts as a barrier to bacteria and immune cells. The ROS-induced loss of barrier function leads to bacterial invasion, local inflammatory responses, and tissue damage. ROS also plays a central role in inflammation. It activates many important molecular events involved in inflammatory reactions, such as NFκb activation and inflammasome formation [7,13,15,17,35]. The prevention of ROS-induced dysfunction of the adherens and tight junction, as well as inflammatory reactions by r-Alb could be the key molecular mechanisms underlying the therapeutic effects.

Of note, in this study, Alb was reductively modified via treatment with DTT. The treatment markedly increased the number of –SH groups in Alb. DTT not only maintained Cys34 at the reduced state but also released more –SH groups through cleaving the intra-chain disulfide bonds. In contrast to normal Alb with only one reactive SH group, r-Alb had more than ten other –SH groups. Furthermore, all of them reacted with H_2_O_2_ and GSSG. Consequently, r-Alb exhibited far more potent antioxidative activity than normal Alb.

Our study could have important implications. First, the results of this study highlight the importance of Alb in body defense against oxidant stress. It challenges the tradition that only emphasizes the importance of the intracellular compartment in cellular protection against oxidants. Second, Alb is one of the well-used bioproducts in clinical practice [19,60]. Pathological situations that require Alb administration, such as cancer, diabetes, rheumatic diseases, liver and kidney diseases, are usually associated with oxidative stress [61]. Administration of r-Alb could be more potent in rebalancing body homeostasis. Third, comparing to other antioxidants, such as catalase and lactoperoxidase used for the treatment of colitis [62], r-Alb has several advantages. It is safe, cheap, readily available, and affordable. It had a strong thiol-antioxidative activity. Moreover, Alb also exerts thiol-independent antioxidative actions through its binding to metals. It also binds to many compounds that are critically involved in the pathogenesis of the diseases, such as LPS [41,54,55,63]. All these factors make r-Alb a better therapeutic option for the treatment of IBD and other oxidative stress-related diseases.

In conclusion, we have revealed, for the first time, that r-Alb attenuates DSS colitis through mechanisms involving –SH groups mediated rebalancing of the systemic redox state. Given that oxidative stress is a common mechanism underlying many life-threatening diseases, r-Alb, acting as a potent antioxidant, could have a wide range of applications.

## Declaration of competing interest

The authors confirm that they have no competing interests.

## Figures and Tables

**figs1:**
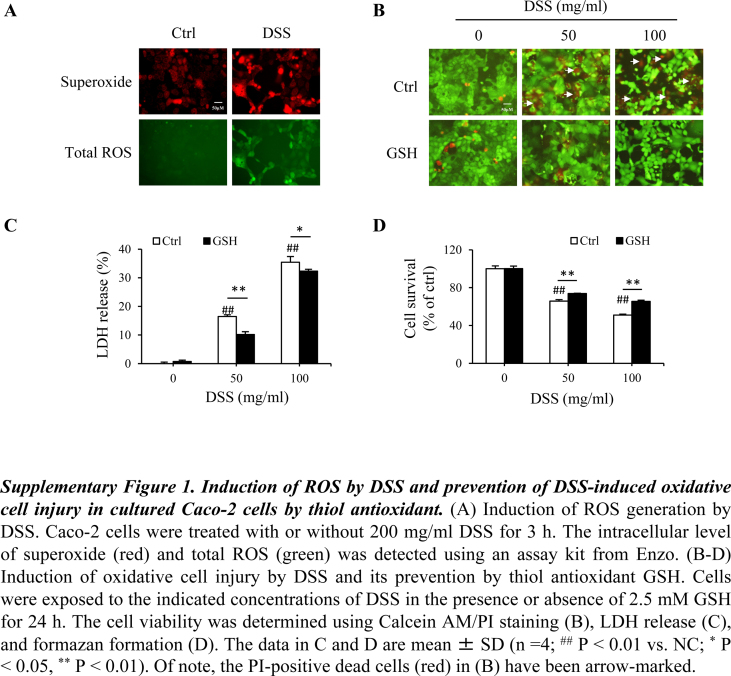


**figs2:**
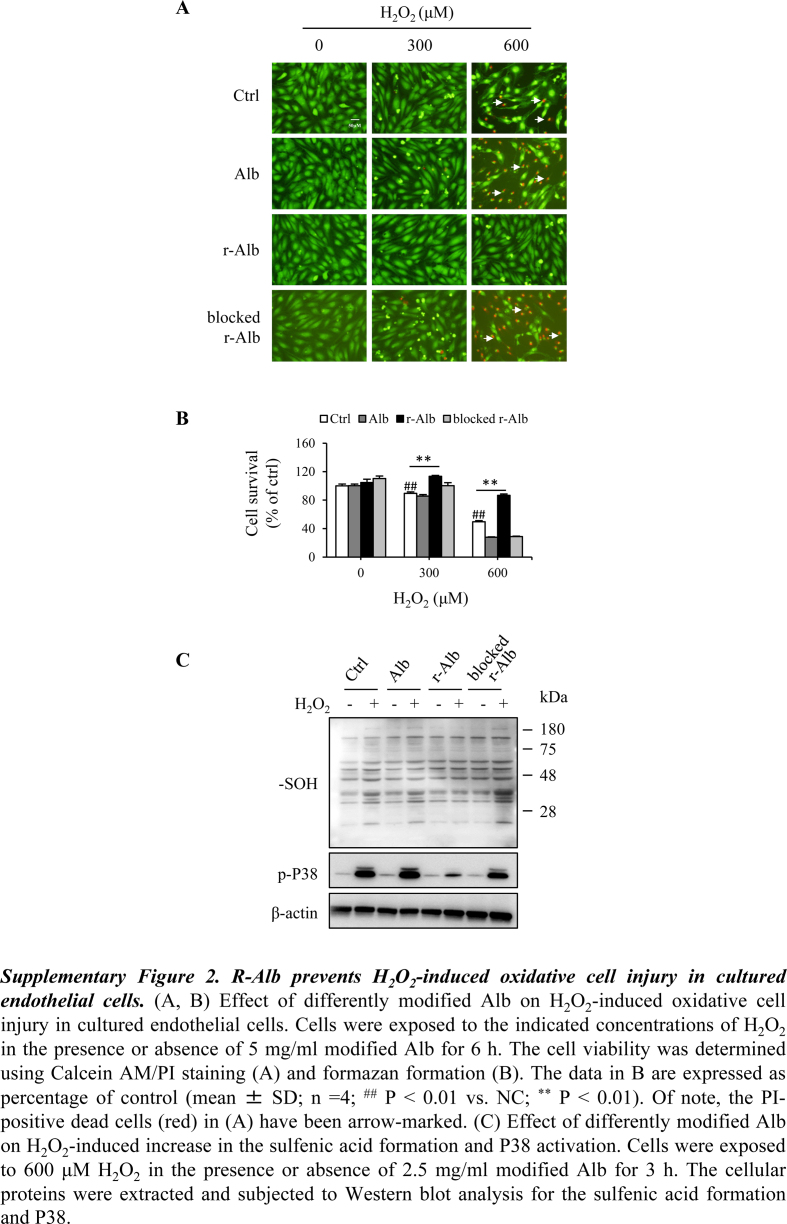


**figs3:**
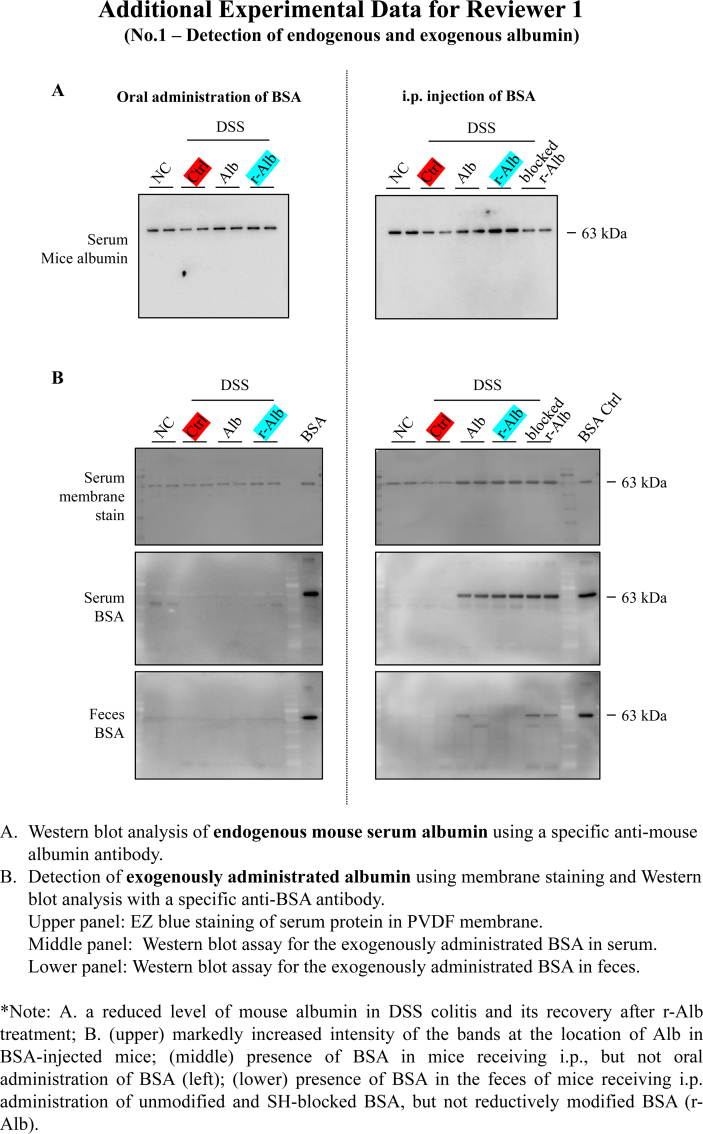


**figs4:**
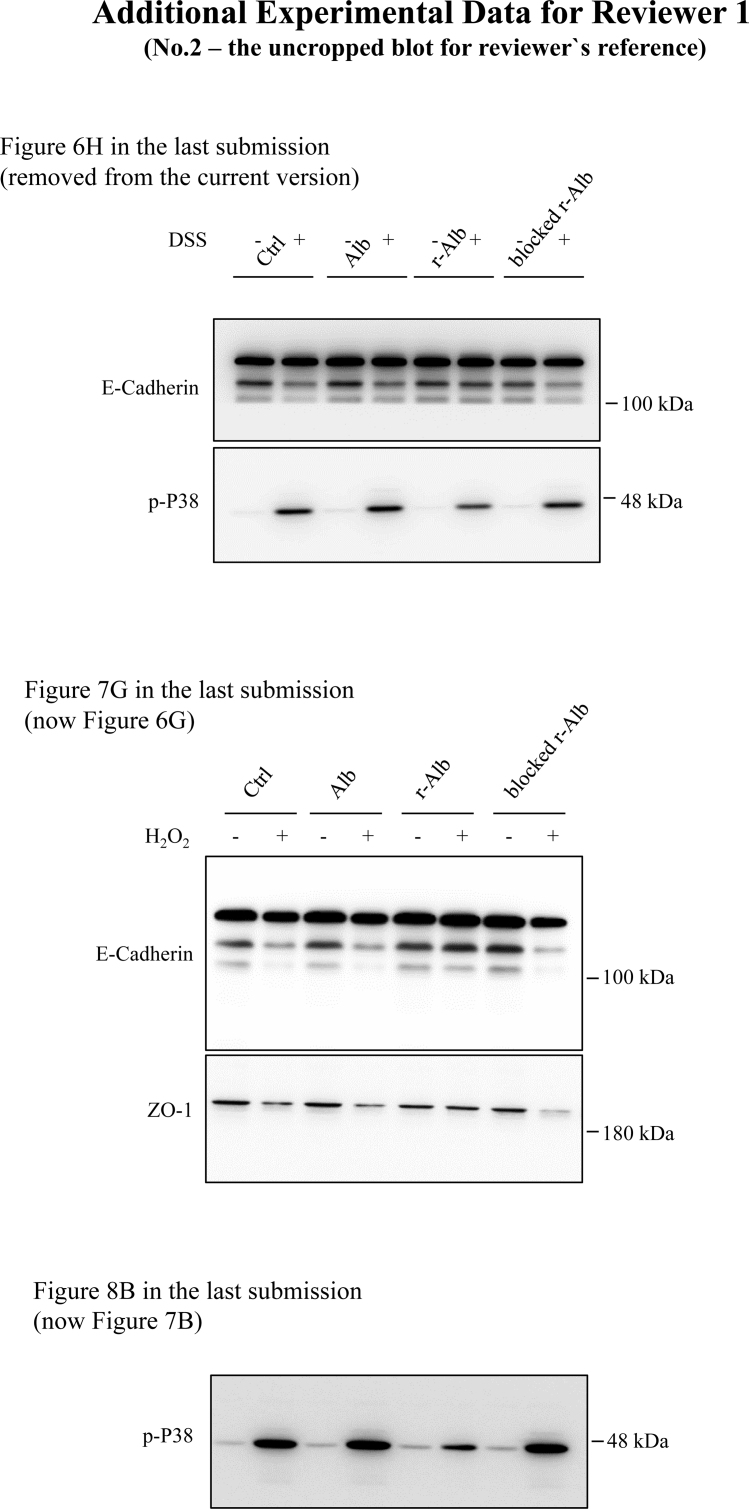


**figs5:**